# Evaluation of Left Heart Function in Heart Failure Patients with Different Ejection Fraction Types using a Transthoracic Three-dimensional Echocardiography Heart-Model

**DOI:** 10.2174/0115734056388350250903130655

**Published:** 2025-09-17

**Authors:** Shen-Yi Li, Yi Zhang, Qing-Qing Long, Ming-Juan Chen, Si-Yu Wang, Wei-Ying Sun

**Affiliations:** 1 Department of Ultrasonography, The People’s Hospital of Hunan Province, The First Affiliated Hospital of Hunan Normal University, Changsha 410005, China

**Keywords:** 3D echocardiography, Cardiac function, Ejection fraction, Heart-Model, Heart failure, LVEF

## Abstract

**Objective:**

Heart failure (HF) is classified into three types based on left ventricular ejection fraction (LVEF). A newly developed transthoracic three-dimensional (3D) echocardiography Heart-Model (HM) offers quick analysis of the volume and function of the left atrium (LA) and left ventricle (LV). This study aimed to determine the value of the HM in HF patients.

**Methods:**

A total of 117 patients with HF were divided into three groups according to EF: preserved EF (HFpEF, EF ≥50%), mid-range EF (HFmrEF, EF =41%–49%), and reduced EF (HFrEF, EF ≤40%). The HM was applied to analyze 3D cardiac functional parameters. LVEF was obtained using Simpson’s biplane method. The N-terminal pro-B-type natriuretic peptide (NT-proBNP) concentration was measured.

**Results:**

Significant differences in age, female proportion, body mass index, and comorbidities were observed among the three groups. With decreasing EF across the groups, the 3D volumetric parameters of the LA and LV increased, while LVEF decreased. The LV E/e' was significantly higher in HFrEF patients than in HFpEF patients. LVEF measurement was achieved in significantly less time with the HM compared with the conventional Simpson’s biplane method. The NT-proBNP concentration increased in the following pattern: HFrEF > HFmrEF > HFpEF. The NT-proBNP concentration correlated positively with LV volume and negatively with LVEF from both the HM and Simpson’s biplane method.

**Conclusion:**

LA and LV volumes increase, and the derived LV systolic function decreases with increasing HF severity determined by the HM. The functional parameters measurements provided by the HM are associated with laboratory indicators, indicating the feasibility of using the HM in routine clinical application.

## 1 INTRODUCTION

Heart failure (HF) is a severe and terminal stage of various cardiovascular diseases. With high mortality and readmission rates, HF generates a large medical burden. The European Society of Cardiology (ESC) guidelines classify HF into three types based on the left ventricular ejection fraction (LVEF): HF with preserved ejection fraction (HFpEF) if LVEF ≥50%, HF with mid-range ejection fraction (HFmrEF) if LVEF 41%–49%, and HF with reduced ejection fraction (HFrEF) if LVEF ≤40% [1]. The etiology, medical history, treatment methods, and prognosis differ among the three types of HF; however, under some conditions, each of the three types can develop into another. For example, HFmrEF can progress to HFpEF or HFrEF, whereas some HFrEF patients can improve to HFmrEF after effective treatment [2]. Therefore, accurate evaluation of LVEF values has important clinical implications.

At present, two-dimensional (2D) echocardiography is the main method used to evaluate left ventricle (LV) function. With conventional echocardiography, the Simpson’s biplane method is applied to measure LV volume and EF in patients with HF. The approach requires obtaining two standard cardiac apical views, is time-consuming, and has certain limitations for application. In pursuit of an improved method, Sun *et al.* developed the transthoracic three-dimensional (3D) echocardiography Heart-Model (HM), which is a deep learning-based automatic evaluation tool for assessing left heart function [3]. The HM avoids the geometric assumptions and apex foreshortening of 2D ultrasound, as well as the complexity and time-consuming nature of manual 3D ultrasound. With the HM, the 3D function of the left atrium (LA) and LV can be quickly and accurately evaluated [3]. However, evidence for the feasibility of using the HM to measure 3D cardiac functional parameters in patients with HF is lacking. To assess the potential clinical value of the HM in patients with HF, this study applied the HM to measure the LA and LV functional parameters in patients with three different types of HF and compared the results with those obtained from the traditional Simpson’s biplane method and NT-proBNP measurements, to affirm the evaluative performance of the HM in HF patients.

## 2 METHODS

### 2.1 Study Population and Design

This study was a prospective observational study that included HF patients who were admitted to the People’s Hospital of Hunan province from January 1, 2021, to January 31, 2024. HF was diagnosed according to the 2021 ESC guidelines [4] based on: symptoms and signs of HF; reduced or normal LVEF; and N-terminal pro-B-type natriuretic peptide (NT-proBNP) >125pg/mL. The exclusion criteria were as follows: age <18 years, history of mechanical valve replacement, congenital heart disease, large amount of pericardial effusion, and low image quality (>2 adjacent segments in a total of 17 segments with poor display of endocardial boundaries). For patients who were hospitalized repeatedly within 1 year, the first hospitalization record was selected. This study was approved by the Ethics Committee of People’s Hospital of Hunan Province (approval number: 2024-203) and was performed in compliance with the Declaration of Helsinki. All participants provided written informed consent.

### 2.2 HM Method

**Fig. (1):**
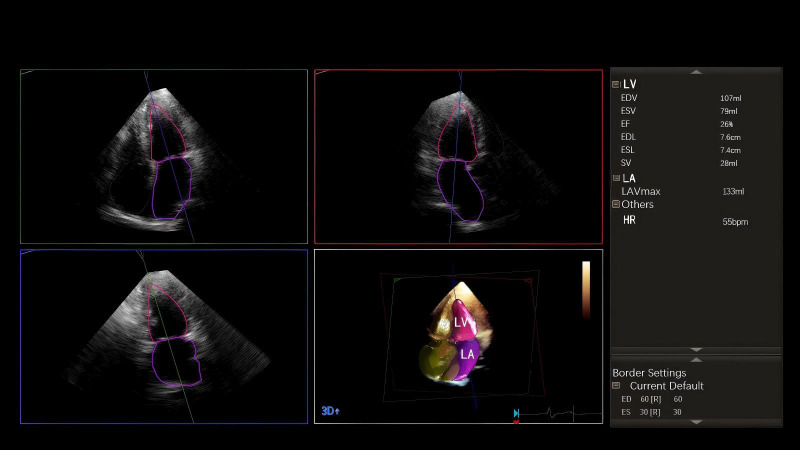
Apical four chamber view for a patient with HFrEF. Under the HM, the software automatically outlines the contours of the LA and LV and then analyzes and displays the following parameters: EDV (end diastolic volume), ESV (end systolic volume), 3D EF (ejection fraction), EDL (end diastolic diameter), ESL (end systolic diameter), SV (stroke volume), LAVmax (left atrial maximum systolic volume), and HR (heart rate).

For application of the HM, a Philips EPIQ 7C color Doppler ultrasound diagnostic instrument (Philips Ultrasound Co., Ltd.) and an X5-1 probe with a frequency of 1–5 MHz were applied. The endocardial boundary values set by the software were 60-30. Patients were placed in the left supine position and connected for electrocardiography (ECG). The LV apical four-chamber view was collected for four consecutive cardiac cycles at the end of a single breath hold. Under the HM, the following parameters were automatically generated: LV end diastolic volume (LVEDV), LV end systolic volume (LVESV), 3D-LVEF, and LA maximum volume (LAVmax) (Fig. **1**). The LA maximum volume index (LAVImax) was calculated as LAVImax = LAVmax/BSA (body surface area). According to the 3D-LVEF, HF patients were divided into three groups: HFpEF (LVEF ≥50%), HFmrEF (LVEF 41%–49%), and HFrEF (LVEF ≤40%). The time required to measure the corresponding parameters was recorded. The echocardiographic examination was completed within 48 hours of hospital admission.

### 2.3 Conventional Echocardiography

With conventional echocardiography, we measured LV end diastolic diameter (LVDD), LV end systolic diameter (LVSD), LV posterior wall (LVPW), inter-ventricular septum (IVS), mitral valve early diastolic peak velocity (E), and the ratio of mitral orifice peak early diastolic blood flow to tissue Doppler mitral annulus peak early diastolic velocity (E/e'). The LV volumetric parameters, including LVEDVSimpson, LVESVSimpson, and LVEFsimpson, were obtained using Simpson’s biplane method. The time required to measure parameters by the Simpson’s biplane method was recorded.

### 2.4 Determination of Serum NT-proBNP

Plasma NT-proBNP concentration was measured using an enzyme-linked immunosorbent assay (Caris200 chemilumi-nescence instrument of Beijing Wantai) for all patients within 24 h of admission.

### 2.5 Statistical Analysis

SPSS (25.0) software was used for data analysis. Data that conformed to a normal distribution are represented as mean ± standard deviation. Analysis of variance was used for inter-group comparisons across the three groups. The least significant difference (LSD) method was used for pairwise comparisons between two groups. Non-normally distributed data are represented as median (1^st^ and 3^rd^ quartiles), and inter-group comparisons were conducted using the Mann–Whitney U test. Categorical data are represented as frequency (%), and inter-group differences were determined using the c^2^ inspection. Correlations were identified using Pearson correlation analysis. Statistical significance was defined by values of *P<*0.05.

## 3 RESULTS

### 3.1 Comparison of Patients' Characteristics among HF Types

Among the 129 HF patients treated during the research period, 7 patients were excluded from this study due to a poor acoustic window, and 5 patients were excluded due to a lack of sufficient breath holding. Thus, a total of 117 patients were ultimately included in the study, with an age range of 45–88 years. The demographic and clinical characteristics of the study population are presented in Table **1**.

Significant differences in age and female proportion were observed among the three groups, with higher values in the HFpEF group compared with the HFmrEF and HFrEF groups (*P*<0.05) and no significant difference between the HFmrEF and HFrEF groups (*P*>0.05). Patient height was greater in the HFrEF group than in the HFpEF group (*P*<0.05), but no significant difference was detected in comparison with the HFmrEF group (*P*>0.05). No significant differences in weight and BSA were found among the three groups, and body mass index (BMI) did not differ between the HFpEF and HFmrEF groups (*P*>0.05). However, the BMI of the HFpEF group was significantly higher than that of the HFrEF group (*P*<0.01), and the BMI of the HFmrEF group was higher than that of the HFrEF group (*P*<0.05). The ratio of concomitant hypertensive heart disease and atrial fibrillation followed the order of HFpEF > HFmrEF > HFrEF, while the ratio of concomitant coronary heart disease and old myocardial infarction followed the order of HFrEF > HFmrEF > HFpEF (*P*<0.05). The ratio of combinations with diabetes did not differ significantly among the three groups (*P*>0.05).

### 3.2 Comparison of Echocardiographic Parameters among HF Types

The 3D LV functional parameters were successfully determined by HM analysis for all patients. When the operator believed that the automatic detection of the LV endocardial surface was not completed correctly, traces of the end diastolic and end systolic phases were manually revised. From the 3D analysis, LVEDV, LVESV, LAD, LAVmax, and LAVImax showed the pattern of HFrEF > HFmrEF > HFpEF, while 3D-LVEF followed the pattern of HFrEF < HFmrEF < HFpEF (*P*<0.05). From the conventional echocardiography, LVDD, LVSD, LVEDV-Simpson, and LVESV-Simpson followed the order of HFrEF > HFmrEF > HFpEF, while LVEF-Simpson followed the pattern of HFrEF < HFmrEF < HFpEF (*P*<0.05). No significant differences in IVS, LVPW, and E were observed among the three groups (*P*>0.05), but the E/e' of the HFrEF group was significantly higher than that of the HFpEF group (*P*<0.05; Table **2**).

### 3.3 Comparison of Three Methods for Measuring EF and Analysis of Time Consumption

**Fig. (2):**
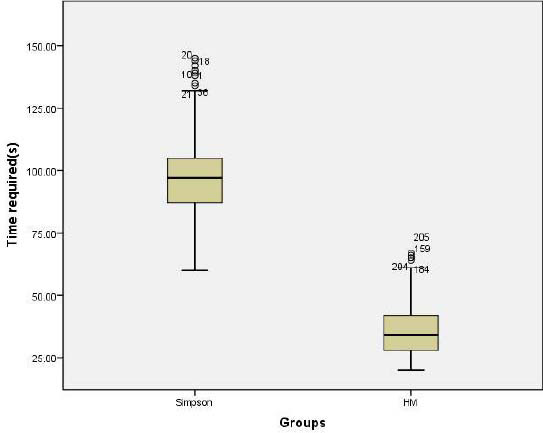
Comparison of the time required to assess LVEF between the HM and Simpson’s biplane method. The X-axis represents the measurement method, and the Y-axis represents the required time. The time required for LVEF analysis was significantly less for the HM compared with the Simpson’s biplane method.

Pearson correlation analysis showed that the 3D-LVEF measured by HM correlated well with the 2D-LVEF measured by conventional two-dimensional echocardiography as well as the LVEF-Simpson measured by the Simpson’s biplane method, with correlation coefficients of 0.953 and 0.904, respectively. The time required to measure LVEF using the HM was significantly less than the time needed using the Simpson’s biplane method (36.77±12.11 s *vs*. 98.15±17.85 s, *P<*0.01; Fig. **2**).

### 3.4 Laboratory Parameter Comparisons

**Fig. (3):**
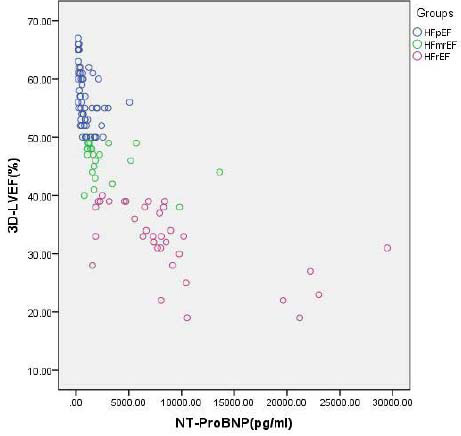
Correlation between 3D-LVEF and NT-ProBNP concentration. The X-axis represents the NT-ProBNP concentration, and the Y-axis represents the 3D-LVEF value. A negative correlation was observed between the 3D LVEF and NT-proBNP concentration in HF patients.

**Fig. (4):**
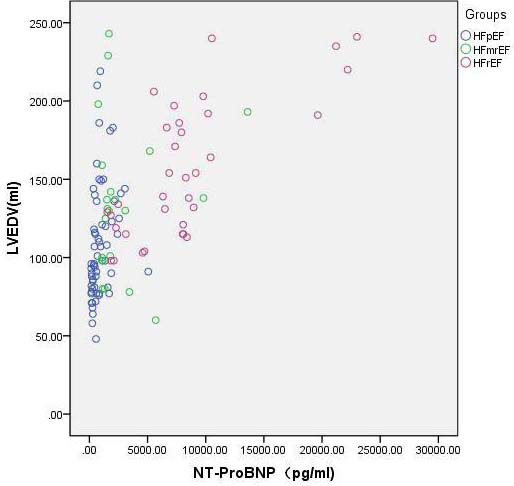
Correlation between 3D LVEDV and NT-ProBNP concentration. The X-axis represents the NT-ProBNP concentration, and the Y-axis represents the LVEDV value. A negative correlation was observed between the 3D LVEDV and NT-proBNP concentration in HF patients.

NT-proBNP concentrations differed significantly among the HF severity groups (*P*<0.05; Table **2**). NT-proBNP concentrations in HF patients were positively correlated with LVEDV and LVEDV-Simpson (r=0.603 and 0.666, respectively) and negatively correlated with 3D-LVEF and LVEF-Simpson (r=-0.735 and -0.670, respectively; Figs. **3** and **4**).

### 3.5 Reproducibility Analysis

The HM analyses were repeated in a randomly selected 30 study participants for intra- and inter-observer variability assessment. The time interval at which the same examiner repeated the assessment was 2 weeks. The calculated intraclass correlation coefficients (ICCs) indicated excellent inter-observer reliability (0.75–0.81%) and test-retest reliability (0.72–0.84%).

## 4 DISCUSSION

### 4.1 Significance of Evaluating Cardiac Function in HF Patients with Differing LVEF

HF is a clinical syndrome caused by abnormal cardiac structure or function leading to impaired ventricular filling or ejection function. Previous studies [5, 6] have revealed differences in the pathogenesis, epidemiology, clinical characteristics, and prognosis of HF with different EF types. HFpEF is primarily characterized by diastolic dysfunction, and its main pathological mechanisms include a systemic inflammatory response, endothelial dysfunction, and intracellular calcium overload. In patients with HFpEF, the active relaxation of the LV is impaired, and myocardial stiffness is increased. In contrast, HFrEF is primarily characterized by systolic dysfunction, and its main pathological mechanisms involve myocardial damage, abnormalities, and remodeling caused by activation of the neuroendocrine system. HFmrEF represents a large group of patients with heterogeneous characteristics, and its prevalence rate among all HF cases is 14–24%. The clinical features of HFmrEF fall between those of HFrEF and HFpEF and can dynamically change from one type to the other. Both systolic and diastolic functions can show some degree of impairment in HFmrEF patients, and the underlying pathological mechanisms remain incompletely understood [7]. HF can change from one type to another over time, and the different categories can have different clinical outcomes after drug treatment as well as different prognoses. Although the molecular mechanisms underlying the transitions among HFrEF, HEmrEF, and HFpEF are largely unknown [8], rapid and accurate assessment of cardiac function can guide clinical intervention in a timely manner, thus preventing disease progression and reducing the occurrence of adverse events such as death.

### 4.2 Clinical Characteristics of Different LVEF-based HF Categories

The clinical characteristics associated with the three types of HF vary, with HFpEF occurring more often among elderly obese individuals and females [9]. Obesity is thought to be a major driver of the pathophysiology of HFpEF, as it can promote alterations in cardiomyocyte signaling pathways as well as myocardial inflammation and fibrosis, leading to myocardial dysfunction and remodeling along with systemic endothelial dysfunction [10]. Menopause-related estrogen dysregulation and the associated comorbidities, such as obesity, diabetes, hypertension, and renal insufficiency, also may contribute to the development of HFpEF, which represents one cause for the high prevalence of HFpEF in postmenopausal women [11]. A prospective multicenter study [12] found that, compared with HFrEF patients, HFmrEF patients were older and included more females. However, compared with HFpEF patients, HFmrEF patients were younger and included more males. HF is often accompanied by multiple complications, with HFpEF being more commonly associated with hypertension and HFrEF more commonly associated with coronary heart disease and myocardial infarction [13]. The eccentric remodeling of the heart in HFrEF patients is significant, while the LA is stiffer in HFpEF patients, making them prone to concomitant atrial fibrillation [14]. Prior research has found no significant differences among the three types of HF in terms of history of percutaneous coronary intervention, hyperlipidemia, diabetes, or anemia [15].

### 4.3 Significance of Measuring 3D Left Heart Function Parameters

Evaluation of LV and LA volumes and function is important for early diagnosis, disease monitoring, and prognosis prediction in patients with cardiovascular disease. The European Association for Cardiovascular Imaging and the American Society for Echocardiography recommend the use of LV volumes measured by 3D echocardiography in clinical practice [16]. 3D-LVEF and 3D-LVESV measured by 3D echocardiography are the strongest predictors of poor prognosis (all-cause mortality or length of hospital stay) in patients, with predictive values exceeding those of functional parameters measured by 2D echocardiography. 3D echocardiography parameters can also be used for accurate HF risk stratification [17]. Rickenbacher *et al.* analyzed the echocardiographic data of a cohort of elderly patients with congestive HF [18] and found that the systolic parameters decreased gradually from HFpEF to HFmrEF and then to HFrEF, while diastolic dysfunction showed no variation among the three groups. A study using spot tracking LV short-axis torsion reported significant decreases in LV torsion/untwisting parameters in HFpEF patients, which were due to a decrease in LV myocardial motility [19]. HF often presents with LV remodeling, including LV enlargement with a tendency towards sphericity and decreased contractile force. With progression from HFpEF to HFmrEF to HFrEF, the 3D volume of the LV cavity gradually increases, and evidence of increased LV filling pressure and LV hypertrophy is common among all three groups of HF [20]. However, the LV in HFrEF patients mainly exhibits eccentric hypertrophy, while the LV in HFmrEF and HFpEF patients mainly displays concentric remodeling (to a lesser extent in the latter condition) [21].

The LA plays a crucial role in heart filling and optimizing cardiac output. LA size is an important indicator for evaluating LV diastolic function, and the LAVmax measured using 3D echocardiography correlates well with CMR imaging results, making it a predictive factor for severe cardiovascular disease [22]. Patients with different EF types have differing LAVmax and LAVImax values, indicating that changes in the LA occur via different pathological mechanisms among the three types of HF. Historically, LA dysfunction and elevated pressure have been considered markers of HFpEF, while HFrEF is typically associated with LV dysfunction [23]. However, in the current study, we observed worse LAVmax and LAVImax values in the HFrEF group compared with the HFmrEF and HFpEF groups, which may be due to the fact that HFrEF patients often have moderate to severe functional mitral regurgitation, which leads to a greater LA burden. Packer *et al.* [24] found that HFrEF is commonly associated with eccentric ventricular remodeling, which is related to mitral valve leaflet abnormalities. Although LA abnormalities are considered a hallmark of HFpEF, the LA structure and function were worse in HFrEF patients than in HFpEF patients. Therefore, the need to understand the intrinsic LA pathological changes should be equally emphasized for both HFrEF and HFpEF patients.

The E/e' can sensitively reflect an increase in LV filling pressure and is not affected by factors such as pre- and after-loads of the ventricle, heart rate, and hemodynamic changes. The E/e' is one of the recommended indicators for evaluating LV diastolic dysfunction [25]. A linear relationship exists between E/e 'and LA pressure. In the present study, the E/e' showed a greater increase in HFrEF patients than in HFpEF patients, indicating a more significant increase in LA pressure in HFrEF patients.

### 4.4 3D Automatic Cardiac Function Technology

The HM is a deep learning-based automatic 3D quantification model used to measure heart parameters through the quick and accurate quantification of the volume and function of the left heart [26]. Measurements produced by the HM are highly correlated with and only slightly deviate from cardiac measurements obtained using CMR imaging and traditional manual 3D ultrasound [27]. The gold standard method for diagnosing HF is CMR. We did not use CMR in the current study due to its high cost and long operation time. However, a good correlation between 3D echocardiography and CMR in cardiac function analysis was reported by previous studies. For example, Nicol *et al.* [28], demonstrated high agreement between the 3D HM and CMR or left ventricular angiography (LVA) when stratifying patients according to HF categories. Consistently, Levy *et al.* [29], observed excellent correlations for ESV and LVEF measurements obtained by the 3D HM and CMR for (r=0.93 and r=0.91, respectively). The HM has also been shown to provide highly repeatable results with low intra- and inter-observer variabilities [20]. The HM can also help diagnose chamber enlargement and irregular wall movement [30]. The HM measures full volume data from 3D echocardiography for a single cardiac cycle, making it suitable for patients with arrhythmias such as atrial fibrillation [31]. LA volumetric assessment specifically can be challenging in clinical practice. Due to the geometric shape of the LA and deviation of its true long axis from the long axis of the LV, 2D ultrasound may not provide accurate LA measurements. The HM represents the first automated technology designed to quantify LA volume from a 3D echocardiographic dataset and thus has the potential to accurately analyze changes in LA 3D volume [32]. Use of the HM also significantly reduces the average time required for LV and LA analysis. Volpato *et al.* showed that the HM is fast and significantly less time-consuming than manual 2D or semi-automatic 3D full-volume acquisition and measurement [33]. Narang *et al.* [27] compared the HM with manual 3D analysis software as well as CMR and found that the HM requires the least amount of time. Our present study compared the time required to apply the HM method for evaluation of cardiac function parameters with the time required to apply the traditional Simpson’s biplane method, and the results demonstrated that the HM is less time-consuming, making it particularly useful in fast-paced clinical settings.

### 4.5 Significance of Laboratory Indicators and their Correlation with Ultrasound Indicators

NT-proBNP is a well-recognized, sensitive indicator of the severity and prognosis of HF that is known to be positively correlated with ventricular wall tension and end-diastolic pressure. The NT-proBNP concentration increases in HF, and previous studies have demonstrated correlations between echocardiographic indicators and the plasma NT-proBNP concentration [34, 35]. Athavale *et al.* [36] showed that in HFrEF and HFmrEF patients, NT-proBNP levels are similarly elevated but are much higher than those in HFpEF patients. However, changes in NT-proBNP concentration are also influenced by many factors and diseases, and thus, its specificity for diagnosing HF severity is limited.

### 4.6 Study Limitations

The acquisition of images for the HM requires careful patient cooperation, and thus, patients who could not cooperate with breath holding were excluded from the study. However, this exclusion criterion may have resulted in a certain degree of selection bias, as these patients may have had severe HF. The cross-sectional design of this study is another important limitation, as is the small sample size. Moderate to severe mitral regurgitation, which is often observed in HFrEF patients, can significantly impact LA volume. Due to the small number of these patients, we did not include the MR factor in the analysis of LA volume. We also did not consider potential disease conditions, such as dilated cardiomyopathy or aortic stenosis, in the analysis of LV volume. This lack of subclassification may have impacted the results of the study, and such conditions need to be further analyzed in future studies with larger sample sizes, which will be a focus of our future research.

## CONCLUSION

Cardiac function parameters measured using the HM show good correlation with laboratory indicators. Application of the HM is clinically feasible and requires less time than the Simpson’s biplane method. Thus, the HM can be recommended for routine application in more medical institutions.

## Figures and Tables

**Table 1:** Comparison of demographic and basal clinical characteristics among the three HF groups based on EF (HFpEF, HFmrEF, and HFrEF).

-	**HFpEF Group** **(n=60)**	**HFmrEF Group** **(n=22)**	**HFrEF Group** **(n=35)**
Age (years)	69.48±8.06	65.27±9.17*	64.71±11.75*
Female [number(%)]	34(56.7%)	7(31.8%)*	7(20%)*
Body height (cm)	161.37±6.39	163.55±7.35	164.77±5.81*
Body weight (kg)	66.55±8.04	67.73±9.62	63.06±8.95
Body surface area (m^2^)	1.69±0.12	1.72±0.14	1.66±0.13
BMI (kg/m^2^)	25.59±3.08	25.42±4.08	23.23±3.12^**#^
Heart rate (bpm)	73.62±11.77	72.00±15.83	76.66±11.85
Hypertension [n(%)]	42(70)	11(50)*	16(45.7)^*#^
Coronary artery disease [n(%)]	29(48.3)	15(68.2)*	26(74.3)^*#^
Prior myocardial infarction [n(%)]	7(11.7)	6(27.3)*	11(31.4)^*#^
Atrial fibrillation [n(%)]	21(35)	6(27.3)	7(20)*
Diabetes [n(%)]	19(31.7)	7(31.8)	9(25.7)

Note:*Compared with the HFpEF group, *P*<0.05, **P<0.01^; #^Compared with the HFmrEF group, *P*<0.05, ^##^*P*<0.01.

**Table 2:** Comparison of cardiac parameters and NT-proBNP concentrations among the three HF groups.

-	**HFpEF group** **(n=60)**	**HFmrEF group** **(n=22)**	**HFrEF group** **(n=35)**
LVEDV (ml)	109.10±37.15	134.31±48.68*	158.26±44.89^**#^
LVESV (ml)	46.93±18.60	73.46±28.95**	108.66±38.31^**##^
3D-LVEF (%)	56.82±5.17	45.77±3.25**	32.37±6.17^**##^
LAD (mm)	34.67±5.11	37.55±6.47*	41.85±6.69^**#^
LAVmax (ml)	55.46±15.34	69.32±22.56**	83.97±28.59^**#^
LAVI (ml/m^2^)	32.96±8.89	40.77±14.37**	50.84±17.60^**#^
LVDD (mm)	48.30±5.50	52.00±7.28*	61.86±10.78^**##^
LVSD (mm)	25.53±4.41	29.56±5.25**	39.09±8.62^**##^
2D-LVEF (%)	60.92±5.28	48.45±3.58**	37.17±4.36^**##^
IVS (mm)	11.16±1.65	11.79±2.35	10.78±1.15
LVPW (mm)	10.69±1.24	10.84±1.27	10.50±1.36
E (cm/s)	63.93±14.63	60.50±10.38	59.00±13.24
E/e '	13.39±6.44	15.66±3.40	17.24±6.57**
LVEDVSimpson (ml)	111.18±26.58	137.00±42.12**	162.51±43.53^**#^
LVESVSimpson (ml)	47.95±17.19	75.05±28.08**	111.86±39.86^**##^
LVEFSimpson (%)	57.58±7.93	46.09±6.55**	32.27±9.37^**##^
NT-proBNP (pg/ml), median (P_25_, P_75_)	607.00 (307.75, 1318.50)	1644.50 (1146.25, 3171.50)**	7910.00 (4730.00,9780.00)^**##^

**Abbreviations:** 2D-LVEF: two-dimensional left ventricular ejection fraction, 3D-LVEF: three-dimensional left ventricular ejection fraction, E: mitral valve early diastolic peak velocity, E/e ': ratio of mitral orifice early diastolic peak blood flow to Tissue Doppler mitral annulus early diastolic peak velocity, IVS: inter-ventricular septum, LAD: left atrial diameter, LAVmax: left atrial maximum volume, LAVI: left atrial maximum volume index, LVDD: left ventricular end diastolic diameter, LVEDV: left ventricular end diastolic volume, LVEDV-Simpson: LVEDV measured by Simpson’s biplane method, LVESV: left ventricular end systolic volume, LVESV-Simpson: LVESV measured by Simpson’s biplane method, LVPW: left ventricular posterior wall, LVSD: left ventricular end systolic diameter, LVEF-Simpson: LVEF measured by Simpson’s biplane method Notes:*Compared with the HFpEF group, *P*<0.05, **P<0.01; ^#^Compared with the HFmrEF group, *P*<0.05, ^##^*P<*0.01.

## Data Availability

The datasets generated and analyzed during the current study are not publicly available due to limitations of ethical approval involving patient data and anonymity, but are available from the corresponding author [Y.Z] on reasonable request.

## LIST OF ABBREVIATIONS

3D-LVEF: Three-dimensional left ventricular ejection fraction
CMR: Cardiac magnetic resonance
E: Mitral valve early diastolic peak velocity
E/e': Ratio of mitral orifice early diastolic and peak blood flow to Tissue Doppler mitral annulus early diastolic peak velocity
IVS: Inter-ventricular septum
LA: Left atrial
LAVI: Left atrial maximum volume index
LAVmax: Left atrial maximum volume
LV: Left ventricular
LVDD: Left ventricular end diastolic diameter
LVEDV-Simpson: Left ventricular end diastolic volume measured by Simpson’s biplane method
LVEDV: Left ventricular end diastolic volume
LVEF-Simpson: Left ventricular ejection fraction measured by Simpson’s biplane method
LVESV-Simpson: Left ventricular end systolic volume measured by Simpson’s biplane method
LVESV: Left ventricular end systolic volume
LVPW: Left ventricular posterior wall
LVSD: Left ventricular end systolic diameter

## STANDARDS OF REPORTING

STROBE guidelines were followed.
